# Radiographic outcomes of stick-assisted manipulation in adolescent idiopathic scoliosis: a retrospective study of 57 patients

**DOI:** 10.3389/fped.2026.1871248

**Published:** 2026-06-26

**Authors:** Xingda Chen, Chenxing Huang, Wanyan Chen, Rueishiuan Jiang, Jiahui He, Zhuangzhuang Tan, Zhaojun Cheng, De Liang, Xiaobing Jiang, Jingjing Tang

**Affiliations:** 1 College of Orthopedics and Traumatology, Guizhou University of Traditional Chinese Medicine, Guiyang, China; 2 Department of Spine Surgery, The Second Affiliated Hospital of Guangzhou Medical University, Guangzhou, China; 3 Shanghai Municipal Hospital of Traditional Chinese Medicine, Shanghai University of Traditional Chinese Medicine, Shanghai, China; 4 The First Affiliated Hospital of Jinan University, Jinan University, Guangzhou, China; 5 Guangzhou University of Traditional Chinese Medicine First Affiliated Hospital, Guangzhou, China

**Keywords:** adolescent idiopathic scoliosis, conservative treatment, physiotherapy, spinal manipulation, the stick-assisted-manipulation

## Abstract

**Background:**

The conservative management of adolescent idiopathic scoliosis (AIS) typically involves protracted treatment cycles; however, conventional modalities articularly exercise therapies often suffer from poor patient compliance due to the heavy burden of prolonged parental supervision. To address these clinical challenges, we propose Stick-assisted manipulation (SAM), a novel physical intervention based on manual therapy. This study aims to evaluate the clinical efficacy and safety of this novel SAM approach in the treatment of AIS, offering a potential solution to overcome barriers in treatment adherence.

**Methods:**

A retrospective analysis was conducted of patients with scoliosis who were treated exclusively with SAM, a manual therapy based physical intervention at our institution between January 2023 and August 2024. Treatment efficacy was assessed by comparing pre- and post-treatment radiographic and clinical parameters, including the Cobb angle, vertebral body rotation (VBR) grade, shoulder height difference (SHD), and coronal balance index (CBI).

**Results:**

After a 1-month treatment course consisting of four SAM sessions, the mean Cobb angle decreased significantly from 18.24° ± 10.16° to 13.10° ± 11.39° (*P* < 0.001). And the Cobb angle improved by ≥6° in 22 of 57 patients (38.60%). Significant improvements were also observed in SHD and VBR grade. No treatment-related complications or adverse events were recorded during the intervention period or throughout post-treatment follow-up.

**Conclusion:**

SAM demonstrates favorable clinical and radiographic outcomes in patients with AIS, with a good safety profile. These findings suggest that SAM may represent a broadly applicable and safe conservative treatment option for adolescent idiopathic scoliosis.

## 1 Introduction

Adolescent idiopathic scoliosis (AIS) represents a significant health concern among adolescents worldwide (1). Current management strategies for AIS are broadly categorized into surgical and non-surgical treatments. In clinical practice, treatment decisions are primarily guided by the magnitude of the coronal Cobb angle measured on standing radiographs: surgical intervention is generally recommended for curves greater than 40°, brace treatment for curves between 20° and 40°, and other conservative approaches for curves less than 20° (2). A large-scale study involving 3,315 patients with more than 10 years of follow-up demonstrated no significant difference in long-term quality-of-life improvement between surgically and non-surgically treated AIS patients (3). Owing to advantages such as being noninvasive, painless, and cost-effective, conservative management is commonly considered the first-line option for AIS patients with Cobb angles below 40° and without evident neurological complications (4).

Conservative treatment modalities for AIS mainly include bracing, therapeutic exercise, and spinal manipulation. Among these, the effectiveness of brace treatment is highly dependent on patient compliance and is frequently associated with psychological distress in adolescents (5). Exercise-based therapy alone has shown limited efficacy in reducing Cobb angles and does not demonstrate a significant protective effect against curve progression exceeding 5°, with the current evidence characterized by substantial uncertainty (6). In contrast, spinal manipulation therapy has been reported to significantly reduce Cobb angles over short-term interventions (e.g., 6 weeks), particularly in mild-to-moderate curves ranging from 10° to 30°, while also improving lower-limb kinematics and postural stability, including gait and stair negotiation (3). While isolated reports, such as a case study on Traditional Chinese medicine manual therapy for adolescent idiopathic scoliosis, suggest that spinal manipulation can be effective, high-quality evidence remains insufficient (7). Existing literature indicates that traditional manipulation still lacks robust proof of independent efficacy, suffers from poor reproducibility, and is restricted by the absence of standardized treatment protocols (8, 9).

In response to these limitations, our research team, drawing on accumulated clinical experience, explored and developed a novel physical therapy approach for AIS: Stick-assisted manipulation (SAM). This technique is characterized by operational simplicity, short intervention duration, and notable therapeutic efficacy, enabling meaningful reductions in scoliotic curvature within a relatively brief treatment period. To further substantiate its clinical utility, the primary objective of the present study was to evaluate the effectiveness, a clinically meaningful reduction (≥6°) in Cobb angle and improvement in vertebral rotation and shoulder balance, and safety of SAM in the treatment of AIS.

## 2 Methods

### 2.1 Participants

We retrospectively reviewed 83 patients with AIS who attended the scoliosis outpatient clinic at our hospital between January 2023 and August 2024. Of these, 57 received SAM treatment. For patients presenting with Cobb angles more than 20°, and particularly those exceeding 40°, the attending physicians routinely recommended adjunctive brace treatment in accordance with established clinical guidelines. However, a proportion of patients declined brace therapy due to factors such as cost, psychological burden, or academic pressure.

### 2.2 Inclusion criteria

(1) A Cobb angle greater than 10°; (2) age between 8 and 18 years; and (3) availability of at least two full-spine radiographic examinations obtained before and after manual therapy.

### 2.3 Exclusion criteria

(1) A history of spinal surgery; (2) the presence of severe systemic diseases affecting other organ systems; and (3) receipt of other conservative treatments during the study period, including exercise therapy, bracing, acupuncture, or similar interventions.

### 2.4 Stick-assisted-manipulation therapy and process

SAM was developed by Professor Jiang Xiaobing's team at the Second Affiliated Hospital of Guangzhou Medical University, based on the application of wooden instruments for treating spinal musculoskeletal disorders within the Lingnan school of traumatology. Guided by the traditional Chinese medicine (TCM) concept of “tendon–bone balance,” SAM adopts a holistic, spine-centered approach with an emphasis on restoring coordination and equilibrium between muscular and skeletal structures.

**Figure 1:**
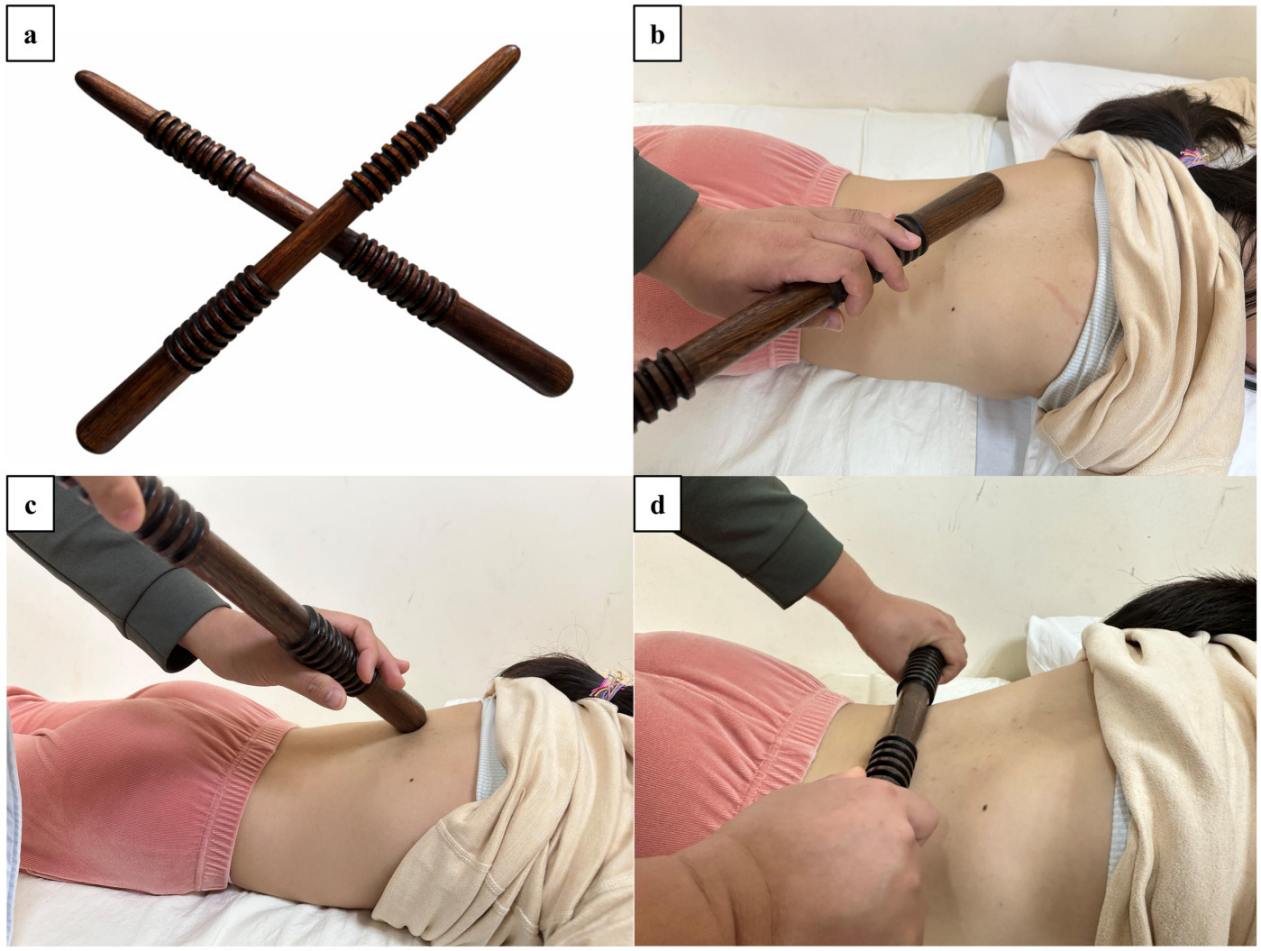
Schematic illustration of stick-assisted massage therapy for adolescent scoliosis. **(a)** The therapeutic stick, consisting of a head, a tip, and a stick body. **(b)** *Cuo* technique. **(c)** *Dian* technique. **(d)** *Gan* technique.

The therapeutic stick used in SAM is supported by multiple patents (ZL 202020903725.2; ZL 201720353760.X and ZL 202130066879.0) (Figure 1a). Compared with conventional manual manipulation techniques, SAM employs the therapeutic stick to simulate and extend the functional capabilities of finger-based acupressure. This allows the intervention to reach treatment depths that are difficult to achieve with standard manual techniques, thereby enabling efficient, noninvasive release of deep-seated musculature and chronically strained or stiff muscles.

Each treatment session lasted 25–30 min and consisted of three sequential manipulation techniques–CUO, DIAN, and GAN–performed using different parts of the therapy stick. The procedure followed a progressive principle from superficial to deeper tissue manipulation. The rounded large head of the stick was used for CUO, the small tip for DIAN, and the cylindrical shaft for GAN. The CUO technique (5–8 min) targeted superficial paraspinal soft tissues. Using the head of the stick, longitudinal rubbing along the direction of muscle fibers and small circular rubbing movements were performed within approximately 3–5 cm lateral to the spinous processes, primarily focusing on the concave side of the scoliotic curve to reduce superficial muscle tension and improve soft-tissue flexibility. The DIAN technique (8–12 min) focused on deeper paraspinal tissues. The tip of the stick was applied to areas of increased muscle tension along the convex side of the spinal curvature. Localized compression, slow traction along the muscle fiber direction, and gentle rotational pressure were applied. Each target point was maintained for approximately 3–5 s, with pressure gradually adjusted according to patient tolerance. The GAN technique (5–8 min) served as a recovery phase. Using the shaft of the stick, gentle rolling movements were applied longitudinally and transversely along both sides of the spine, combined with mild rotational and oscillatory movements to promote muscle relaxation and local circulation. SAM was administered once per week for four consecutive weeks (total of four sessions). The treatment procedure is shown (Figures 1b–d; Supplementary File).

### 2.5 Outcome measurements

The basic data of the subjects, including age, sex, and body mass index (BMI), were collected before and end the treatment. Other parameters included full-length spine radiographs, all data were collected and organized by two associate chief physicians (spinal surgeons) who were blinded to the treatment status and clinical outcomes. Discrepancies >3° were resolved by a third independent assessor. The full-length spine radiographs were measured to obtain the corresponding Cobb angle, Risser sign, vertebral body rotation (VBR) grade and alignment of the C7 plumbline (C7PL) in relation to the central sacral vertical line (CSVL) (C7PL-CSVL) (10–13). C7PL deviates more than 2 cm vertically to the left or right of CSVL in the coronal plane, indicating coronal plane imbalance (CPI) (14). A vertical shoulder height difference of more than 1 cm between both sides is defined as shoulder height discrepancy (SHD) (15).

### 2.6 Data analysis

All statistical analyses were performed using SPSS version 25.0 for Windows (IBM Corp., Armonk, NY, USA). The normality of continuous variables was assessed using the Shapiro–Wilk test. Categorical variables are presented as percentages, while continuous data are expressed as mean ± standard deviation $\left(\bar{x}\pm s\right)$. Within-group comparisons were conducted using paired-sample *t* tests. Between-group differences were analyzed using paired-sample *t* tests, *χ*^2^ test or the One-Way ANOVA test, as appropriate. A two-sided *P* value <0.05 was considered statistically significant.

## 3 Results

**Figure 2:**
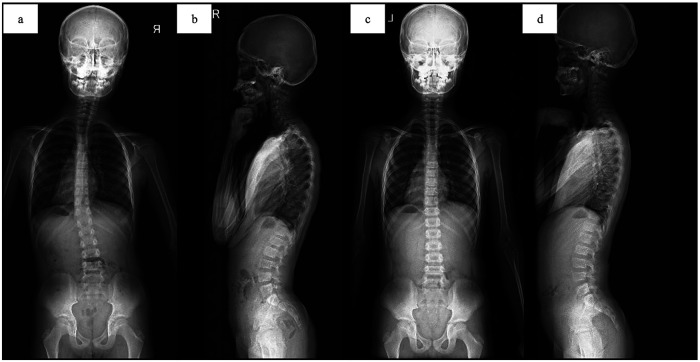
A typical case. The patient is a 10-year-old male participant, first diagnosed on July 28, 2023. x-Ray images show **(a,b)** the upper end vertebra at T7, the lower end vertebra at L3, with an initial Cobb angle of 15.32° and leftward scoliosis. The shoulder height difference was 19.69 mm, with the apex of the curve at T11, coronal balance offset at −18.59 mm, and vertebral rotation at grade 1. Sagittal balance was not recorded. Risser sign was grade 0, and the pelvic triangular cartilage state was grade 1. The patient underwent 4 sessions of rod-point treatment over a period of 1 month. Follow-up on September 1, 2023, showed **(c,d)** a Cobb angle reduced to 0.09°, shoulder height difference of 0 mm, coronal balance of 0 mm, and vertebral rotation remaining at grade 1.

A total of 83 patients with AIS who received SAM were initially screened. Of these 26 patients were excluded due to incomplete pre- and post-treatment radiographic data, failure to complete the full treatment course, or other related reasons. Ultimately, 57 AIS patients were included in the final analysis, comprising 31 males and 26 females. The mean age of the included patients was 15.62 ± 0.65 years, and the mean BMI was 17.94 ± 0.93 kg/m^2^. Regarding the distribution of Cobb angles, 75.4% of patients had a main curve between 10° and 20°, 21.1% had a main curve between 20° and 40°, and 3.5% presented with a main curve greater than 40°. Left-sided main curves were observed in 59.6% of patients, while 40.4% exhibited right-sided curves. Most patients (59.6%) had a Risser sign of grade 4 or 5, indicating that the majority of individuals treated with SAM were in the late stages of skeletal maturity (Table 1). A typical case has been provided (Figure 2).

**Table 1:** Demographic characteristics of the participants.

Characteristics	Number (%)/MD ± SD
Gender	
Male	31 (54.4%)
Female	26 (45.6%)
Age (years)	15.6 ± 0.7
BMI (kg/m^2^)	17.9 ± 0.9
Primary curve	
Cobb 10–20°	43 (75.4%)
Cobb 20–40°	12 (21.1%)
Cobb >40°	2 (3.5%)
Primary curve direction (left/right)	34 (59.6%)/23 (40.4%)
Risser sign grade	
0	7 (12.2%)
1	4 (7%)
2	3 (5.2%)
3	2 (3.5%)
4	19 (33.3%)
5	22 (38.5%)

BMI, body mass index.

Comparisons of radiographic parameters before and after treatment demonstrated that SAM led to significant improvements across multiple outcomes. As the primary indicator of scoliosis severity, the Cobb angle was significantly reduced following SAM therapy, decreasing from 18.24° ± 10.16° before treatment to 13.10° ± 11.39° after treatment (*P* < 0.001). SHD also showed a significant improvement, with 42 patients demonstrating correction after treatment (*P* = 0.038). In contrast, CPI did not exhibit a significant change after one month of treatment (*P* = 0.678). In addition, the distribution of VBR grades shifted markedly toward lower grades following treatment, indicating a significant reduction in axial rotation (*P* < 0.001) (Table 2).

**Table 2:** Participants’ pre- and post-treatment parameter assessment.

Parameters	Pre-treatment	Post-treatment	95% CI	*t*/*χ*^2^	*P*
Cobb°	18.2 ± 10.2	13.1 ± 11.4	3.663–6.630	6.952	**<0** **.** **001**
SHD (N/Y)	32/25	42/15	–	4.300	**0** **.** **038**
CPI (N/Y)	26/31	51/6	–	0.408	0.678
VBR grade (0/1/2/3/4)	0/36/18/5/0	5/35/15/2/0	–	76.483	**<0** **.** **001**

SHD, shoulder height discrepancy; VBR, vertebral body rotation grade; CPI, coronal plane imbalance; N, no; Y, yes. Bold values indicate statistical significance (*P* < 0.05).

A responder analysis was performed to evaluate the proportion of patients achieving a clinically meaningful Cobb angle reduction. Using a threshold of ≥6°, which exceeds the established minimal clinically important difference of 5°–6° (16, 17), 22 of 57 patients (38.60%) demonstrated significant improvement after the 4-week SAM course. Stratified by baseline severity, 17 of 43 patients (39.53%) in the Cobb 10°–20° group and 4 of 12 patients (33.33%) in the Cobb 20°–40° group achieved ≥6° improvement. In the Cobb >40° group 1 of 2 patients (50.00%) met the responder criterion (Table 3).

**Table 3:** Participants’ outcome after stick-assisted manipulation.

Parameters	No Cobb angle improvement (<6°)	Cobb angle improvement (≥6°)
Cobb 10–20°	26 (60.47%)	17 (39.53%)
Cobb 20–40°	8 (66.67%)	4 (33.33%)
Cobb >40°	1 (50.00%)	1 (50.00%)
Total	35 (61.40%)	22 (38.60%)

Subgroup analyses comparing male and female patients revealed a mean change in Cobb angle of 4.92° ± 5.61° in males and 5.39° ± 5.45° in females, with no statistically significant difference between sexes (*P* = 0.755). Similarly, no significant sex-based differences were observed in SHD, CPI, or VBR before and after treatment, suggesting that SAM exerts comparable therapeutic effects in both male and female AIS patients (Table 4).

**Table 4:** Comparison of pre- and post-treatment effects between male and female.

Parameters	Male	Female	95% CI	*t*/*χ*^2^	*P*
ΔCobb°	4.9 ± 5.6	5.4 ± 5.5	−3.412 to 2.490	−0.313	0.755
Pre-SHD (N/Y)	16/15	16/10	–	0.566	0.452
Pre-CPI (N/Y)	12/19	14/12	–	1.306	0.253
Pre-VBR grade (0/1/2/3/4)	0/20/10/1/0	0/16/8/2/0	–	0.566	0.820
Post-SHD (N/Y)	20/11	22/4	–	2.946	0.086
Post-CPI (N/Y)	27/4	24/2	-	0.408	0.678
Post-VBR grade (0/1/2/3/4)	1/21/1/0/0	4/14/6/2/0	-	5.403	0.151

SHD, shoulder height discrepancy; VBR, vertebral body rotation grade; CPI, coronal plane imbalance; N, no; Y, yes.

## 4 Discussion

Present, both domestic and international treatment strategies for AIS are predominantly conservative in nature, encompassing brace treatment, exercise therapy, physical therapy, and manual interventions. Among these, bracing remains the most widely used conservative approach; however, it is associated with several well-recognized limitations, including skin irritation and pressure ulcers, muscle atrophy and decreased muscle strength, as well as potential psychological burden and deterioration in quality of life with long-term use (18). Moreover, the effectiveness of brace treatment is strongly dependent on daily wearing time (19), yet many adolescents fail to achieve the prescribed duration, thereby compromising therapeutic outcomes (20). Exercise-based interventions similarly face challenges related to suboptimal patient adherence (21).

Physical therapy approaches for AIS are diverse, and the literature generally categorizes these interventions into two broad types: soft tissue–oriented release techniques and joint correction–based manipulative techniques, which are typically applied in combination during clinical practice (22). Some studies have reported that manual therapy alone does not confer significant benefits in Cobb angle reduction when compared with minimal intervention, and that meaningful therapeutic effects are observed only when manual techniques are combined with other conservative modalities such as bracing or exercise therapy (5, 9). In addition, Fusco et al. suggested that exercise and physical therapy interventions may need to be sustained for at least six months to achieve clinically relevant improvements (23). Consequently, identifying a conservative treatment option that is safe, efficient, and associated with good patient compliance remains a key clinical challenge in the management of AIS.

As a noninvasive physical therapy modality, SAM demonstrated favorable efficacy and safety in the treatment of AIS in the present study. Following SAM therapy, significant improvements were observed in the Cobb angle, VBR, and SHD, with no treatment-related adverse events recorded. In the current literature, achieving a structural curve regression of ≥5° or 6° remains a significant challenge for conventional conservative modalities. Recent studies indicate that the true curve improvement rate (regression ≥5° or 6°) for rigid bracing typically ranges from 20.3% to 29.2% (24, 25). Similarly, high-quality RCTs evaluating Schroth exercises have reported an improvement rate of only 16% (6), with recent meta-analyses highlighting that the mean correction often fails to reach the 5° clinical threshold (26). In contrast, our retrospective cohort demonstrated that 38.6% of patients treated with the SAM protocol achieved a clinically significant improvement of ≥6°. While our single-arm design precludes claims of direct superiority, these comparative historical data strongly suggest that SAM is a highly promising candidate for inducing active curve regression, warranting further validation through rigorous randomized controlled trials.

The plasticity of scoliotic deformities decreases markedly in the late stages of skeletal development (27). In the present cohort, a subset of patients had already reached late skeletal maturity (Risser grades 4 or 5), yet SAM still produced meaningful radiographic and functional improvements in this population. This finding indicates that SAM may retain therapeutic value even in adolescents with advanced skeletal maturity, thereby offering a novel conservative treatment option for this subgroup. Furthermore, subgroup analyses revealed comparable treatment effects between male and female patients, as well as generally consistent responses among patients with mild to moderate scoliosis, underscoring the cross-sex applicability and overall stability of SAM as a therapeutic intervention.

From a mechanistic perspective, previous studies have demonstrated that asymmetrical activity of the paraspinal muscles is a key contributor to the onset and progression of AIS, particularly during the early stages of the disease (28). Patients with AIS typically exhibit increased muscle activation and hypertrophy on the convex side of the curve, whereas muscle differentiation on the concave side is often impaired, with reduced strength accompanied by fatty infiltration and structural degeneration. This imbalance generates a persistent lateral traction force that promotes curve progression (29–32). In addition, neurological factors such as proprioceptive deficits and abnormalities in postural control may also contribute to disease development by influencing paraspinal muscle function (33).

Based on these pathological characteristics, we propose that SAM may exert its therapeutic effects in AIS through multiple complementary mechanisms. First, by applying targeted mechanical stimulation to hypertonic muscles and myofascial trigger points on the convex side of the curve, SAM may reduce abnormal muscle tension and suppress asymmetric electromyographic activity (34). Second, the release of deep fascial adhesions may decrease the sustained lateral forces acting on the spine, thereby improving the biomechanical environment for vertebral growth. Third, the sensory input generated during manual manipulation may, through central nervous system plasticity, enhance proprioceptive feedback and neuromuscular coordination, ultimately facilitating the recovery of postural control (35). Nevertheless, the precise biomechanical and neuroregulatory mechanisms underlying these effects require further elucidation through basic and translational research.

Unlike conventional conservative treatments (such as rigid bracing or specific exercises) which demand long-term, highly active, and subjective daily participation from pediatric patients, SAM is a short-term (4-week), doctor-led, passive physical intervention. Because it requires minimal active effort from the children and has a strictly defined, brief treatment cycle, we clinically observed an inherently higher patient compliance.

In summary, SAM represents a safe, effective, and highly compliant conservative intervention for AIS. It appears applicable across sexes, varying levels of skeletal maturity, and different severities of spinal curvature, and therefore holds considerable promise for widespread clinical use in the conservative management of AIS.

Several limitations of this study should be acknowledged. First, the sample size was relatively small, and patient-reported outcome measures, such as quality-of-life or functional assessment questionnaires, were not collected before and after treatment. Consequently, the present findings lack a comprehensive evaluation of patients' subjective perceptions and psychosocial responses to SAM therapy. Second, the proportion of patients with severe AIS included in this cohort was limited; therefore, the therapeutic efficacy of SAM in patients with severe scoliosis requires further investigation in studies with larger sample sizes. In the future, we will design and conduct an RCT trial for the treatment of AIS with SAM, and carry out long-term follow-up.

## 5 Conclusion

In conclusion, this study demonstrates that SAM, as a noninvasive physical therapy modality, is safe and effective in the conservative management of AIS. Following short-term intervention, SAM significantly improved the Cobb angle, vertebral body rotation, and shoulder height difference, with no treatment-related adverse events observed, indicating favorable clinical feasibility. Furthermore, SAM demonstrated stable and consistent efficacy across sexes, supporting its broad applicability and reproducibility.

## Data Availability

The raw data supporting the conclusions of this article will be made available by the authors, without undue reservation.
