# Supplementary material for: Radiographic outcomes of stick-assisted manipulation in adolescent idiopathic scoliosis: a retrospective study of 57 patients
Source: Front Pediatr. 2026 Jun 26;14:1871248. doi: 10.3389/fped.2026.1871248 (PMC13349910; doi:10.3389/fped.2026.1871248)
Supplement: Supplementary file 1 [file Supplementaryfile1.docx]

**Standard Operating Procedure of Stick-Assisted Manipulation for Adolescent Idiopathic Scoliosis**

Chapter 1: General Provisions

1.1 Objectives

This manual is formulated to standardize the clinical operations of Stick-Assisted Manipulation (SAM) in the treatment of Adolescent Idiopathic Scoliosis (AIS), ensure the safety, efficacy, and consistency of the intervention, enhance the quality of clinical care, minimize the occurrence of adverse events, and provide standardized technical guidance for spinal specialists. Based on the Traditional Chinese Medicine (TCM) theories of soft tissue injury (Jinshang) and bone-setting (Zhenggu), and integrated with modern spinal biomechanics as well as cumulative clinical experience, this manual is applicable to the departments of spinal surgery and orthopedics across all levels of medical institutions conducting relevant clinical work.

1.2 Scope of Application

This manual applies to pediatric patients aged 8 to 18 years with a confirmed diagnosis of Adolescent Idiopathic Scoliosis (AIS) who meet the following criteria: a Cobb angle greater than 10°; in the progressive or stable stage of the disease; with or without symptoms such as bilateral muscle asymmetry, muscle spasms, pain, or restricted spinal mobility; and presenting no contraindications for manual therapy. This manual serves strictly as an operational standard for Stick-Assisted Manipulation and does not cover other clinical management stages, such as diagnostic evaluation, rehabilitation training, or surgical indication assessment.

1.3 Core Principles

Principle of Treatment Based on Syndrome Differentiation (Bian Zheng Shi Zhi): The manipulation protocol should be individualized and dynamically tailored based on the patient's specific scoliosis curve type, magnitude (Cobb angle), muscle status, and constitutional characteristics.

Principle of Equal Emphasis on Soft Tissue and Bone Structure (Jin Gu Bing Zhong): Soft tissue regulation facilitates structural realignment ("regulating soft tissues to rectify the bone structure"). Through stick-assisted manipulation, spastic muscles are relaxed, meridians are cleared, and the spinal alignment line is adjusted, thereby simultaneously achieving soft tissue repair and structural spinal correction.

Principle of Gentleness and Precision: The manipulation centers on gentle, deep-penetrating, and precise forces, strictly avoiding violent impacts to protect the developing spinal bones, joints, and surrounding soft tissues of adolescents.

Principle of Safety First: Contraindications must be rigorously evaluated before the operation; the patient's real-time responses must be closely monitored during the procedure; and timely follow-ups must be conducted post-treatment to proactively prevent various complications.

Chapter 2: Pre-operative Assessment and Preparation

2.1 Patient Assessment

2.1.1 Clinical Diagnosis Verification

Verify the patient's historical diagnostic records and imaging data, including full-spine anteroposterior (AP) and lateral radiographs, CT, or MRI findings. Clearly clarify the specific scoliosis curve type (e.g., thoracic, lumbar, or thoracolumbar/combined curves), Cobb angle magnitude, degree of vertebral rotation, and the presence or absence of concomitant kyphotic or lordotic deformities. Non-idiopathic types, such as congenital scoliosis, neuromuscular scoliosis, and metabolic disease-associated scoliosis, must be strictly excluded.

2.1.2 Suitability Assessment for Manual Therapy

Symptomatic Assessment: Inquire about the presence of neck, shoulder, low back, or back pain, limb numbness, weakness, or dyspnea. Evaluate pain intensity using the Visual Analogue Scale (VAS) and locate the specific sites and severity of muscle spasms.

Physical Signs Assessment: Conduct a thorough clinical examination via inspection (observing the symmetry of the shoulders, scapulae, and hips, and the alignment of the spinal midline), palpation (assessing the alignment of the spinous processes, paraspinal muscle tension, and the presence of tenderness or fibrotic nodules/cord-like bands), and range of motion (ROM) evaluation (measuring spinal flexion, extension, bilateral lateral flexion, and rotation) to accurately localize the affected segments and soft tissue involvement.

Screening for Contraindications: This manipulation is strictly contraindicated in patients presenting with any of the following conditions: organic spinal lesions including fractures, dislocations, tumors, tuberculosis, or active infections; coagulation disorders; localized skin breakdown, ulcers, or active inflammation; fever, acute infectious diseases, or severe hepatic/renal insufficiency.

2.1.3 Formulation of Individualized Treatment Protocols

Determine the manipulation intensity, frequency, and course of treatment dynamically based on the patient's age, sex, curve magnitude, affected segments, muscle status, and general constitution. The primary target areas and auxiliary techniques should be clearly delineated. For mild scoliosis (Cobb angle 10°–20°), the protocol focuses primarily on soft tissue regulation (Lijin), supplemented by bone-setting (Zhenggu). For moderate scoliosis (Cobb angle 20°–40°), equal emphasis is placed on both soft tissue regulation and bone-setting, integrated with bracing and specific rehabilitation exercises.

2.2 Preparation of Therapeutic Equipment and Environment

2.2.1 Preparation of Therapeutic Equipment

Stick-Assisted Tools: Select medical-grade wooden material, with a length of 30–40 cm and a diameter of 1.5–2 cm. Both ends must be polished into a blunt, rounded shape with a smooth surface free of burrs. Wipe and disinfect the tools with 75% ethanol before use.

Auxiliary Equipment: A treatment bed (width: 60–80 cm, with adjustable height), disposable medical sheets, medical alcohol pads, and massage media (e.g., medical petrolatum or safflower oil, selected dynamically based on the patient's skin condition).

2.2.2 Environmental Preparation

The treatment room must be kept clean, quiet, and well-ventilated, with the room temperature maintained at 22–26°C to prevent the patient from catching a cold. A disposable medical sheet must be laid on the treatment bed to ensure a sterile and comfortable clinical environment, minimizing external interference to facilitate the practitioner's operation and maximize patient cooperation.

2.3 Practitioner and Patient Preparation

2.3.1 Practitioner Preparation

The practitioner must wear a clean work uniform, a mask, and a cap, and must thoroughly wash and disinfect both hands. The practitioner should be fully familiar with the patient's clinical condition and the designated treatment protocol, clarifying key operational points and potential risk factors. The purpose, procedure, potential discomforts, and precautions of the manual therapy must be informed to the patient and their guardians in advance to obtain formal informed consent.

2.3.2 Patient Preparation

Patients should wear loose, breathable cotton clothing to facilitate the adequate exposure of the spinal region, and remove items such as necklaces, jade pendants, and mobile phones to avoid disrupting the operation. Prior to treatment, patients should empty their bladder and bowels, maintain a relaxed emotional state, cooperate with the practitioner for positioning, and communicate their real-time sensations, avoiding muscle stiffness caused by anxiety, which may compromise the therapeutic efficacy.

Chapter 3: Standard Operating Procedures

3.1 Operational Positioning

Select the appropriate posture based on the specific scoliosis site and the operational stage. The core principle is to fully expose the affected segments, relax the paraspinal musculature, and optimize the practitioner's force application.

Prone Position: Applicable to thoracolumbar and lumbar scoliosis. The patient lies prone on the treatment bed with both arms naturally resting on both sides of the body, and the head turned to one side. The pillow height should be adjusted for comfort to avoid cervical spine compression. A soft pillow (thickness: 3–5 cm) is placed under the chest and abdomen respectively to maintain the spine in a fully relaxed state.

Sitting Position: Applicable to cervical and upper thoracic scoliosis. The patient sits upright on a chair with the back straight, shoulders relaxed, and the head maintained in a neutral position. The practitioner stands behind the patient to perform the manipulation.

3.2 Operational Steps (Step-by-step implementation, total duration: 20–30 minutes/session)

3.2.1 Step 1: Cuo (Friction/Stroking) (5–8 minutes)

Objective: To release back muscle tension and clear the meridians.

Global Relaxation: The practitioner stands positioned beside the patient. Using the broad end of the therapeutic stick, apply a circular friction maneuver (Mo-fa) over the patient's back, lumbar, and gluteal regions. The force should gradually increase from light to heavy, comprehensively covering the paraspinal musculature on both sides of the spine. Continue for 3–5 minutes to alleviate muscle tension and promote local blood circulation.

Localized Relaxation: Target the specific affected scoliotic segments and surrounding musculature using the narrow end of the stick. Focus the friction on key muscles including the erector spinae, trapezius, latissimus dorsi, and quadratus lumborum. For areas presenting with obvious tenderness or fibrotic nodules/cord-like bands, appropriately increase the pressure. Treat each specific site for 1–2 minutes to break down muscle adhesions.

3.2.2 Step 2: Dian (Point-Pressing) (8–12 minutes)

Objective: To stimulate the Bladder Meridian, the Governing Vessel (Du Mai), and specific muscle attachments using the stick, further releasing spastic muscles and restoring soft tissue biomechanical balance. The narrow end of the stick serves as the contact point. The applied force should progressively deepen to penetrate the underlying tissues, strictly avoiding superficial skin abrasion.

Meridian Stick-Pressing: Apply point-pressing along the Bladder Meridian and Governing Vessel on both sides of the spine. Proceed inferiorly from the Dazhui acupoint (GV 14) down to the bilateral sides of the coccyx. Maintain pressure for 3–5 seconds at each specific acupoint (e.g., Dazhui, Fengmen, Feishu, Xinshu, Ganshu, Pishu, Shenshu), repeating the sequence 2–3 times to clear the meridians and harmonize Qi and blood.

Muscle Stick-Pressing: For hypertonic muscles on the convex side of the curve, apply point-pressing along the direction of the muscle fibers. For instance, target the trapezius and rhomboids on the thoracic convexity, and the erector spinae and quadratus lumborum on the lumbar convexity. Conversely, for the hypotonic (lax) muscles on the concave side, apply gentle point-pressing stimulation to facilitate muscle contraction, thereby rebalancing the bilateral muscle tension. Treat each affected segment for 2–3 minutes, ensuring the force penetrates deeply to relieve spasms and release mechanical tension.

Trigger Point Stick-Pressing: Apply localized, sustained pressure to tender points identified during palpation. The intensity should elicit a tolerable sensation of soreness and distension (Deqi sensation), strictly avoiding acute sharp pain. Press each point for 1 minute and repeat twice to resolve local adhesions and alleviate pain.

Para-Spinous Process Stick-Pressing: The practitioner stands on one side of the patient, grasping the body of the stick with the right hand. The left hand holds the stick approximately 8–10 cm above the narrow end, with the left thumb abducted against the patient's skin. The web space (purlicue) of the left hand serves as a stabilized fulcrum at the skin contact point. Using the right hand to generate force, press the stick against the para-spinous region on the convex side of the affected segment. Instruct the patient to relax, then gently lever and rotate the spinous process from an inferior to superior direction to correct the vertebral rotational deformity. The angle of rotation must be strictly guided by the patient's spinal mobility and tolerance, avoiding excessive torque that could induce joint injury. Perform 1–2 repetitions per segment.

3.2.3 Step 3: Gan (Rolling/Smoothing) (5–8 minutes)

Objective: To soothe the treated musculature, alleviate any mild soreness induced by the manipulation, and consolidate the therapeutic efficacy.

Procedure: The practitioner utilizes the body of the stick to perform a continuous rolling motion over the treated muscles. The applied pressure should be exceptionally gentle, smoothly covering the entire treatment zone for 1–2 minutes to enhance local microcirculation and relieve muscle fatigue.

3.2.4 Step 4: Finalization and Post-Treatment Care (2–3 minutes)

Assist the patient in rearranging their clothing and wiping away any residual lubrication medium from the skin.

Postural Adjustment: Assist the patient in changing positions slowly to prevent orthostatic dizziness. Inquire about the patient's post-treatment sensations and closely observe for any adverse or uncomfortable symptoms.

Clean and thoroughly disinfect the therapeutic stick in preparation for the next operational session.

Chapter 4: Post-treatment Observation and Care Management

4.1 Immediate Post-treatment Observation

Following the completion of each session, the practitioner must closely observe the patient's complexion and mental status, inquire about any uncomfortable symptoms such as dizziness, nausea, limb numbness, or exacerbated pain, and inspect the treated site for skin erythema, swelling, or localized breakdown. Mild soreness or distension is considered a normal physiological response, and patients should be reassured that it will resolve spontaneously. In the event of abnormal signs—such as severe pain, persistent limb numbness, or restricted spinal mobility—subsequent treatments must be suspended immediately. A comprehensive physical examination should be performed, supplemented by necessary radiographic or advanced imaging to clarify the underlying etiology and implement appropriate clinical management.

4.2 Home Care Guidance

Posture Management: Instruct patients to maintain correct sitting, standing, and sleeping postures, strictly avoiding round shoulders (slouching), single-shoulder backpacking, and crossing legs. When sitting, the back must be kept straight with a lumbar support pillow placed behind the lower back. For sleeping, the supine or lateral position is recommended, while the prone position must be avoided. The mattress should feature moderate firmness, and the pillow height should be aligned with the width of the shoulder.

Physical Exercise: Guided by the specific scoliosis curve type, prescribe targeted rehabilitation training, such as wall-standing exercises, core stabilization training (e.g., planks or prone back extensions / "flying swallow" exercises), and specific scoliosis corrective exercises. Patients should train for 15–20 minutes daily, adhering to a progressive principle while strictly avoiding over-exercising.

Nutritional Adjustment: Advise patients to increase their dietary intake of foods rich in calcium, vitamin D, and high-quality protein (such as milk, eggs, fish, and soy products) to support skeletal development. Spicy, raw, or cold foods should be limited to optimize general constitution.

Daily Contraindications: Strenuous sports (such as basketball, football, and high jump) and heavy lifting must be avoided to minimize axial spinal loading. Patients should also keep warm and avoid cold exposure at the treated sites to prevent inducing muscle spasms.

4.3 Follow-up Management

Short-term Follow-up: Within 24 hours after each treatment session, follow up with the patient via telephone or WeChat to monitor symptom progression and check for any discomfort. Address queries from patients and their guardians, and dynamically adjust the home care plan if necessary.

Mid-term Follow-up: Upon the completion of each treatment course, repeat full-spine anteroposterior (AP) and lateral radiographs to measure the Cobb angle and evaluate the structural curve correction. Concurrently, perform a physical examination to adjust the subsequent manipulation parameters (such as force intensity, frequency, and primary target segments).

Long-term Follow-up: For patients achieving symptom relief and structural curve stabilization post-treatment, conduct long-term follow-up every 3–6 months for a total duration of 1–2 years. This long-term monitoring tracks spinal skeletal development to proactively prevent curve recurrence, with rehabilitation protocols adjusted as warranted.

Chapter 5: Prevention and Management of Complications

5.1 Common Complications

Under the strict premise of adhering to these standardized operating procedures, the incidence of complications associated with this manipulation is exceptionally low. Potential transient complications may include localized skin erythema or swelling, exacerbated tenderness, increased muscle spasms, temporary limb numbness, or mild dizziness.

5.2 Preventive Measures

Rigorously Execute Pre-operative Assessment: Screen for all contraindications meticulously to avoid implementing manual therapy on unsuitable or ineligible patients.

Master Advanced Manipulation Techniques: Practitioners must possess a thorough and precise understanding of spinal anatomy to avoid mislocalization or inappropriate force application during the procedure.

Closely Monitor Real-time Responses: Continuously observe the patient's feedback during the operation to dynamically adjust the intensity and technique. If the patient experiences significant pain or acute discomfort, suspend the manipulation immediately.

Standardize Equipment Disinfection: Ensure strict implementation of tool disinfection protocols to completely eliminate cross-infection, and provide comprehensive post-treatment home care guidance to minimize external triggers for complications.

Avoid Arbitrary Protocol Modifications: Follow a gradual and step-by-step progression principle. Practitioners must never utilize violent or forceful impacts in pursuit of short-term corrective outcomes.

5.3 Management of Complications

Skin Erythema and Swelling: If skin erythema or swelling occurs due to excessive manipulation force or friction, immediately suspend the operation at the affected site. Clean the local area with lukewarm water, apply medical moisturizing cream, and advise the patient to avoid scratching. The symptoms typically resolve spontaneously within 1–2 days. In subsequent sessions, adjust the force intensity and increase the utilization of the lubrication medium.

Exacerbated Pain or Muscle Spasms: This is frequently induced by over-stimulation or acute patient anxiety/tension. Immediately apply gentle kneading methods (Rou-fa) to relax the localized musculature, supplemented by hot compresses. Instruct the patient to rest and reduce physical activity. If necessary, prescribe topical or oral non-steroidal anti-inflammatory drugs (NSAIDs). Resume the therapy only after symptoms alleviate, with carefully downgraded manipulation force.

Limb Numbness or Dizziness: This may result from temporary nerve compression during the operation or improper positioning. Immediately assist the patient in adjusting their posture, maintaining the head in a neutral position, and resting in a recumbent position. Massage the distal extremities to facilitate blood circulation. Symptoms usually resolve within several minutes to an hour. If the numbness persists, cervical or spinal radiographic/advanced imaging must be performed to rule out structural nerve injury. Suspend manual therapy and initiate targeted symptomatic management.

Chapter 6: Quality Control and Training Requirements

6.1 Quality Control Standards

Operational Standardization: Strictly adhere to the operational workflows delineated in this manual. Ensure accurate patient positioning, and verify that the intensity, frequency, and coverage of the manipulation meet the standardized thresholds without omitting any operational steps.

Efficacy Evaluation Criteria: Upon completion of one treatment course, the quality standard is met if the patient achieves pain relief (a reduction in VAS score of ≥3 points), improvement in spinal range of motion (ROM), and post-treatment radiographs demonstrating a Cobb angle reduction of ≥5°, or stabilization with no further curve progression.

Safety Standards: No severe complications occur during the treatment period. The incidence rate of mild, transient discomfort must be ≤ 5%, and all such instances must be promptly managed and resolved.

Patient Satisfaction: The satisfaction rate of patients and their guardians regarding the treatment process, clinical outcomes, and home care guidance must be ≥90%.

6.2 Practitioner Training Requirements

Qualifications: Practitioners must possess a valid medical license, have ≥3 years of clinical experience in spinal surgery or orthopedics, be fully conversant with spinal anatomy and TCM soft tissue injury (Jinshang) theories, and master the physiological characteristics of adolescent spinal development.

Training Curriculum: The curriculum encompasses the operational workflows of this manual, comprehensive knowledge of scoliosis diagnosis and management, hands-on training in manipulation techniques, prevention and management of complications, and patient-practitioner communication skills.

Assessment and Certification: Upon completion of the training, practitioners must pass both a theoretical examination (maximum score: 100, passing threshold: ≥80) and a practical skills evaluation using simulated patients (maximum score: 100, passing threshold: ≥85). Independent execution of this manipulation is authorized only after successful certification.

Continuous Education: Regularly participate in academic conferences and clinical skills workshops relevant to scoliosis diagnosis and management. Practitioners must dynamically update their knowledge base and continuously optimize their technical manipulation skills based on clinical practice to enhance overall healthcare quality.

Chapter 7: Supplementary Provisions

7.1 Manual Revision

This manual shall be revised biennially (every 2 years) based on clinical practice feedback, advancements in medical technology, and the latest updates to clinical guidelines. Revisions shall be organized and evaluated by a panel of departmental experts, and will be formally implemented upon review and final approval by the hospital's medical administration department.

7.2 Disclaimer

This manual serves strictly as a clinical technical guideline and is exclusively applicable to eligible patients with Adolescent Idiopathic Scoliosis (AIS). Any adverse consequences arising from inherent individual patient variations, non-standardized operational execution by practitioners, patient non-compliance with the treatment, or failure to strictly adhere to the home care guidance shall be borne by the respective responsible parties.

7.3 Date of Implementation

This manual shall formally come into effect on the date of its official promulgation.

Compiled by: The Second Affiliated Hospital of Guangzhou Medical University
